# The Importance of Follow-Up Visits for Children at Risk of Developmental Delay—A Review

**DOI:** 10.3390/diagnostics14161764

**Published:** 2024-08-13

**Authors:** Roksana Malak, Ada Kaczmarek, Brittany Fechner, Włodzimierz Samborski, Jacek Kwiatkowski, Oskar Komisarek, Maria Tuczyńska, Magdalena Tuczyńska, Ewa Mojs

**Affiliations:** 1 Department and Clinic of Rheumatology, Rehabilitation and Internal Medicine, Poznań University of Medical Sciences, 61-701 Poznań, Poland; blemus001@gmail.com (B.F.); wlodzimierz.samborski@ump.edu.pl (W.S.); 2 Department of Clinical Psychology, Poznań University of Medical Sciences, 61-701 Poznań, Poland; adakaczmarek.1999@o2.pl (A.K.); ewamojs@ump.edu.pl (E.M.); 3 SSC of Maxillofacial Orthopaedics and Orthodontics, University of Medical Sciences, 60-812 Poznań, Poland; jacekkwiat@poczta.onet.pl; 4 Department of Plastic, Reconstructive and Aesthetic Surgery, Collegium Medicum in Bydgoszcz, Nicolaus Copernicus University in Torun, 85-821 Bydgoszcz, Poland; oskarkomisarek@gmail.com; 5 SSC of Clinical Physiotherapy, Poznań University of Medical Sciences, 61-701 Poznań, Poland; maria.tuczynska25@gmail.com; 6 Department of Social Sciences and the Humanities, Poznan University of Medical Sciences, 61-701 Poznań, Poland; tuczynska.m@gmail.com

**Keywords:** infants, psychomotor development, follow-up visit

## Abstract

Structured follow-up visits should be accessible for children at risk for developmental delay. Follow-up visits should include a serial neuromotor assessment in the first two years of life (e.g., 3–6, 12, 24 months corrected age), which are repeated during the transition to school. The diagnosis of neuromotor development may be prognostic for important skills later in life. The early diagnosis of a child’s general movements can be helpful in planning appropriately for proper treatment and intervention. These diagnostic assessments should be conducted by qualified healthcare professionals. The evaluation of neuromotor developmental health is specified in the national guidelines and funded by either a national government or public or private healthcare insurance and based on standardized assessment scales. The aim of this study is to show what elements of follow-up visits are recommended. Objectives: The group of patients for whom the structured follow-up systems are intended were children born very preterm (<32 weeks gestation) or full-term born children with severe neonatal complications. Material and methods: The methods for monitoring neurodevelopment include the following: The General Movements Assessment (GMA), the Ages and Stages Questionnaire (ASQ-3), the Bayley Scales of Infant and Toddler Development (BSID-4), and the Parent Report of Children’s Abilities-Revised (PARCA-R). Results: The results of follow-up visits should be registered. Conclusions: The benefits of follow-up neuromotor development assessments can be observed at school age and even in adulthood.

## 1. Introduction

Human development is shaped both by nature and nurture. Human development in the first few years of life is exceptionally rapid across almost all domains, such as social, emotional, cognitive, language, and motor development [1]. The National Resource Center For Health and Safety In Child Care and Early Education advises professionals to perform screening assessments on infants to improve their development [2]. According to the Swiss National Cohort, thanks to follow-up care, 63% percent of very preterm infants do not have any cognitive or psychomotor impairments by the time they reach school age [3].

Several countries have decided to support the psychomotor development of children systemically, not only due to ethical reasons but also due to socioeconomic status. The program for Transmural Developmental Support for Very Preterm Infants and Their Parents in the Netherlands has demonstrated positive outcomes on a child’s development until 5 years of age. This is why Transmural Developmental Support for Very Preterm Infants and Their Parents has established a foundation of legitimacy for implementation [4]. Additionally, a comparative analysis of data from this program, involving 90 infants in the intervention group and 85 infants in the control group, revealed saved costs due to fewer referrals to paramedical professionals, reduced hospital readmissions, and a lower incidence of child abuse during the first year of life in the intervention group compared to the control group (10% vs. 22%, 29% vs. 40%, and 1.2% vs. 6.4%, respectively). In a Business Case, savings in healthcare provision were compared to the estimated costs of the Transmural Developmental Support for Very Preterm Infants and Their Parents program. However, the Business Case’s political will and positive results were fundamental to establishing structural financial support for implementing this program, showing that the savings outweigh the cost. The cost of healthcare provision was calculated in EUR [5].

The European Foundation for the Care of Newborn Infants (EFCNIs), a collaborative organization comprising over 220 healthcare professionals, parents, and industry specialists from more than 30 countries, has created the European Standards of Care for Newborn Health (ESCNH)—a set of standards for newborn care from hospital to school age [6]. These guidelines aim to ensure the best possible care for newborns across Europe. Each country that participates in implementing these standards has its own national guidelines, protocols, and laws (depending on the local situation) that refer to ESCNH. The most vulnerable group included is very preterm infants (<32 weeks’ gestation) or children with severe developmental complications [5]. Therefore, these standards have been developed for the following:

1. Full-term infants who require intensive care.
2. Infants born before 32 + 0 weeks’ gestation.
3. Infants born after 32 + 0 gestation who have or had one or more significant risk factors, such as
  ○ A brain lesion in neuroimaging that is likely associated with developmental problems or disorders (for example, grade 3 or 4 intraventricular hemorrhage or cystic periventricular leukomalacia);
  ○ Grade 2 or 3 hypoxic ischaemic encephalopathy in the neonatal period;
  ○ Severe fetal growth restriction (microcephalic—symmetric growth);
  ○ Severe social or family problems with safety issues for the child;
  ○ Neonatal bacterial or viral meningitis/encephalitis [7].

Another group of patients who benefit from follow-up care are children who have hypothermia [8], congenital heart diseases [9] and chromosomal abnormalities [10].

It is important to address several factors of infant development, including growth, feeding, general health, hearing, visual abilities, speech, cognition, behaviors, and motor skills. If necessary, interventions such as family-centered developmental support, physiotherapy, speech therapy, dietetics, occupational therapy, and psychological support should be provided. According to the National Guideline Alliance, screening assessments should be conducted at 3, 6, 9, 12, 24 (corrected age), and 48 months of age [11,12,13] (Table 1). 

## 2. Infants at Risk of Acquiring Developmental Disabilities

In cases where a child presents with symptoms such as excessive head lag, persistent fisting of the hands beyond four months of age, stiffness or tightness in the legs, posturing, persistent primitive reflexes, an asymmetry of movement, motor delay, or inharmonic gross motor milestones that appear to be acquired excessively early on or in an unusual sequence (such as rolling by arching their back at the age of 1 month and the lack of fidgety movement in the third month of age) cerebral palsy (CP) may be suspected [19,20]. These clinical signs may indicate potential motor dysfunction and should be evaluated by a pediatric neurologist or other healthcare provider with experience in diagnosing and managing CP. Recognizing and diagnosing CP as early as possible in patients is essential to initiating appropriate interventions and optimizing health outcomes. This is possible, especially in the USA, because early intervention programs are not only available for preterm infants but for all infants and are available in every state and territory of the USA [21]. Early intervention is critical in supporting the healthy development of infants and toddlers with developmental delays and disabilities [22]. Moreover, the Centers for Disease Control and Prevention recommend that “If you, your child’s doctor, or other care provider is concerned about your child’s development, ask to be connected with your state or territory’s early intervention program to find out if your child can get services to help” [23]. Similarly, in Switzerland, due to continuous medical care, if clinicians see a symptom of CP, a child is seen earlier than the third month of life [24].

Infants with motor delays have restricted opportunities to interact with their environment and possess limited abilities to learn through action [1]. Current evidence shows that a child’s brain has more potential for neuroplasticity early in life, which may encourage reorganization after perinatal injury. Early therapeutic interventions can help reduce neuromotor conditions and enhance cognitive and motor abilities for infants at risk of developing a neuromotor delay [25]. One particular program, called Coping with and Caring for Infants with Special Needs (COPCAs), was developed in the Netherlands in the early 2000s, specifically for infants with a high physiological risk for developing neurological disabilities [26]. This intervention focuses on teaching parents how to stimulate infant development during daily routines. Physiotherapists treat family members as active and responsible partners in the intervention process. The objective is to expand the child’s range of physical activities and improve their ability to adapt their movements under different circumstances [26,27]. Randomized controlled trials have shown that COPCA’s coaching strategies are effective in infants with a high risk of developing neurodevelopmental disorders [28,29,30,31]. It was found that encouraging infants with CP at 18 months of age to independently engage in motor behaviors through the repetition of trial-and-error experiences and caregiver coaching as elements of the COPCA intervention was beneficial for improving their motor developmental outcomes compared to standard physiotherapy care [28,29,30,31]. A study conducted by Van Balen et al. (2019) revealed that infants participating in the COPCA program exhibited higher abilities to imitate typical development, which was linked to a higher level of overall anticipatory activation at 18 months of age [32]. This was consistent with another randomized controlled trial from 2021 that found that the COPCA program was associated with greater motor outcomes in infants born before 32 weeks than standard infant physiotherapy [26].

The aim of this study is to present recommendations from follow-up visits and the group of individuals who will benefit most from the monitoring of their neuromotor development. The other purpose of this study is to show the role of follow-up visits across almost all continents.

## 3. Materials and Methods

A literature review was performed from January to March 2024. The Preferred Reporting Items for Systematic Reviews and Meta-Analyses (PRISMAs) guidelines were applied to evaluate the methodological quality of the manuscripts [33]. Computer-based searches of the following medical and public databases were performed: PubMed, Medline Complete, Science Direct, Scopus, and Web of Science. Guidelines were published by the European Foundation for the Care of Newborn Infants, the National Institute for Health and Care Excellence (NICE), the American Academy of Pediatrics (AAP), the National Guideline Alliance, the Australian Children’s Education and Care Quality Authority, and the Japan Environment and Children’s Study [34,35,36,37]. These recommendations were divided into the context of standards described in medical articles [38]. The following combinations of keywords were used to search for the intervention of interest: “preterm infant + follow-up consultations”, and “preterm infant + follow up”.

The following inclusion criteria were included in this review: (1) Identification: studies conducted on preterm infants; (2) Screening: systematic and periodic early intervention until school age with no restrictions on the pathogenesis and etiology of prematurity; (3) Eligibility: reviews of the full text excluding any inappropriate studies; and (4) Inclusion: studies must investigate the effects of follow-up screening and early intervention.

Exclusion criteria were as follows: (1) Identification: studies not conducted on preterm infants, including animal models; (2) Screening: a lack of systematic, periodic follow-up visits until the school age (3) Eligibility: no full text and language other than English used, and (4) Indication: the lack of identification of the effects of follow-up visits.

The Grading of Recommendations, Assessment, Development, and Evaluations (GRADE) system was used [39]. These recommendations were divided into high- and moderate-quality categories. Weak-quality recommendations were not considered. 

The term “high quality” means that a specific recommendation is unlikely to be changed due to the number of articles and audit reports showing supporting evidence of it [40]. Papers that show the application of these recommendations are often randomized trials and the quality of the results and evidence presented are high. Moreover, these articles are deemed high quality since they refer to reliability, compliance with best practices, potential benefits for patients, and efficacy in achieving the intended goals. In medicine, the term “high quality” indicates that a particular service or practice meets the highest professional standards and is safe, effective, and beneficial for the population. “High quality” also means that further studies with a high confidence level do not change the conclusions drawn from the presented research.

The term “Moderate quality” indicates a moderate level of certainty that newer studies will not alter the conclusions of regarding the direction of the presented effects; however, new research may change the conclusions regarding the magnitude of this effect. On the other hand, it combines the certainty of evidence with the effect size of evidence and shows interventions [41]. This recommendation is mostly based on prognostic and prospective studies, randomized controlled trials, and systematic reviews where weaker diagnostic criteria and reference standards were presented [40]. To the best of our knowledge, we avoided using any studies that had been used as evidence for this type of recommendation and showed that methodological flaws were inconsistent or provided indirect evidence [41] (Table 2).

## 4. Results

**Figure 1:**
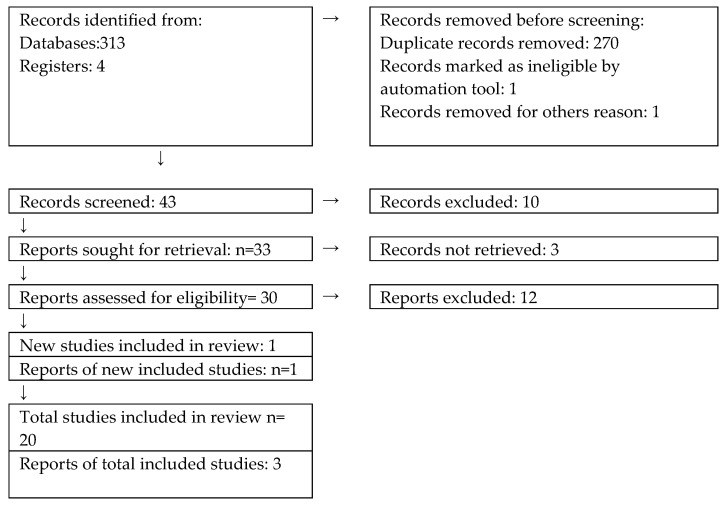
The selection process referring to PRISMA from [33].

We reviewed and analyzed 313 papers. After including the GRADE and PRISMA guidelines, we came up with 23 results (Figure 1).

Next, we rejected “very low”, “low”, and “moderate” recommendations and focused our findings on those that were based on The National Guideline Alliance, The European Foundation for the Care of Newborn Infants, and the American Academy of Pediatrics (AAP). From our search, 20 papers included the following high-quality findings, as presented in Table 3.

## 5. Discussion

Follow-up visits are essential for monitoring the psychomotor development of these preterm infants. These visits should include preterm children born before 32 weeks of gestation and full-term born children who are at risk of developmental disorders, such as brain lesions; stage II or stage III hypoxic-ischemic encephalopathy (HIE) in the neonatal period; severe fetal growth restrictions; severe social or family problems with safety issues for the child; and neonatal bacterial or viral meningitis/encephalitis. Therefore, knowing, in general, that preterm children are at risk for developing motor delays, intellectual disability, visual and hearing impairments, attention-deficit concerns, impulsivity, and hyperactivity, the NICE has developed relevant recommendations, which have been made mandatory in many countries such as Switzerland, the Netherlands, and the US [13,26,55]. These recommendations state that infants who are born before 32 weeks of gestation or those who are born between 32 and 36 weeks of life with specific and identifiable risk factors should receive supplementary support and monitoring until they turn two years old. Infants born at or before 28 weeks of gestation are provided with support and monitoring until they are four years old without any age adjustments. This is due to their heightened risk of having special educational requirements [13]. 

Assessments should be administered periodically since, nowadays, specialists can detect children at risk of CP from a very early age, for instance, before 3–4 months of life and before the transition from preschool to primary school [56]. This is very important because parents often have to make the difficult decision about which school to choose for their child to attend. Some families who have chosen mainstream schools have observed that their children achieve many practical skills, such as literacy, in contrast to those who attend special schools. The greatest benefit for families is a higher level of independence for their children. Even when a brain lesion has been identified in perinatal life, later problems may not appear due to the neuroprotective role of IFCDC, but mostly only if a more independent-oriented therapeutic approach is utilized [57,58,59]. Applying the principles of IFCDC in the long term may reduce the adverse effects of brain lesions. Brain lesions in perinatal life may lead to CP in five to nine percent of preterm children [26]. Studies from the current multicenter study CP-EDIT (Early Diagnosis and Intervention Trial) showed that improving the care of patients with CP is possible even before they have an established diagnosis [60]. The standardized motor assessment scales are an essential component for the early diagnosis of CP. 

In 2018, a comprehensive systematic review and meta-analysis was conducted to provide an overview of the prevalence of CP and motor and cognitive delays in very preterm (VPT) and very low-birthweight (VLBW) infants born within the past decade. This has enabled practitioners to show congruent abnormal findings indicative of CP. Their role is to approach diagnostic-specific early interventions to optimize infant motor and cognitive plasticity, prevent secondary complications, and enhance caregiver well-being [12,58]. Most of the studies included in the analysis evaluated motor and cognitive development at approximately 24 months of age. The most commonly used outcome measure was the BSID scale. Interestingly, the third edition of this scale showed a lower combined prevalence of motor and cognitive delays than the second edition. The findings from this paper revealed that taken as a whole, nearly one in six very preterm infants and one in five VLBW infants had cognitive or motor delays according to developmental scales at around 24 months of age, with approximately 1 in 15 developing CP [59]. 

In another big cohort study conducted by Pascal et al. (2020), trained physiotherapists and educational psychologists assessed preterm infants’ (<31 weeks’ gestation and or/<1500 g birthweight) motor, cognitive, and language performance using the BSID-III scale. The study found that at 2 years of corrected age, 25.2% of VPT and VLBW infants had mild NDI, while 10.9% had moderate-to-severe impairment. CP was diagnosed in 4.3% of the patients. However, in the subgroup of infants born at less than 26 weeks of gestational age, over 60% were found to have developed nephrogenic diabetes insipidus by 2 years of age, highlighting the significance of consistent follow-up care for a correct diagnosis, especially in the most vulnerable of infants. This clinical study concluded that lower gestational age and birthweight are linked to increased rates of adverse neurodevelopmental outcomes [60].

The studies that presented these results used scales such as the Bayley Scales of Infant and Toddler Development (BSID), or ASQ, which means that many aspects of development were considered (i.e., motor, cognitive, social) and focused on the family, not just on the child [55,56]. The WHO and The European Foundation for the Care of Newborn Infants have also recommended using PARKA since the COVID-19 pandemic. Using standardized tools of assessment enables a practitioner to register each patient, periodically compare their results, analyze the results, and also consult on their state with an appropriate specialist, even if the specialist is far away from the center where the follow-up visit takes place [57]. The children who attend follow-up visits present fewer cognitive and psychomotor problems, even at school age [3]. 

Follow-up visits should be conducted in a structured way that is standardized and family-friendly. Therefore, there is no need to create new assessment tools because those that already exist and are available are effective, as shown in many studies. Countries that would like to provide follow-up consultations should base their plans on those countries that have already conducted follow-up consultations for many years, such as Norway, Belgium, Switzerland, and the USA. 

Unfortunately, little is known about early diagnosis in 50% of all CP cases, which are only discernible later in infancy. This is why follow-up visits should also be performed in late infancy, the toddler stage, and before the transition to school. Regular monitoring during this period is essential for detecting developmental coordination disorder (DCD), attention-deficit/hyperactivity disorder (ADHD), autism spectrum disorder (ASD), and other specific learning disorders [61,62,63,64]. An early proper diagnosis enables a child and their family to obtain relevant support and teaches the child practical skills that they can use in their future life. 

Follow-up visits should be accessible, routinely revised, consistent, funded, and supported. It seems unethical not to conduct these assessments because it is generally acknowledged that preterm children are at risk of many problems that can be detected early. Proper interventions should be conducted, which can then positively impact these children in school and later in adulthood [65].

Limitations of this study include the lack of a Polish version for the follow-up visit. The reason why it was not described in the article is the need to show only those follow-up visits that are based on structured, standardized, and validated scales presented by such organizations as The European Foundation for the Care of Newborn Infants, The National Institute for Health and Care Excellence (NICE) and the American Academy of Pediatrics (AAP). The aim of this article is to show what follow-up visits look like in most countries, where they bring many benefits to a population, including social, economic, and certainly health advantages. 

## 6. Conclusions

This study summarizes how important it is to conduct follow-up consultations for children at risk of developmental delay in order to further widen the application of this intervention across several countries. Follow-up visits for preterm infants are critically important for their development. A follow-up visit helps a practitioner and family plan for the appropriate therapy for children at risk of motor developmental delay as well as children with a diagnosis of cerebral palsy. Many benefits of the regular assessment of preterm children have been proven clinically, and therefore, an early assessment allowing for early intervention is obligatory in many countries. The way regular, systematic, periodic patronage visits are supposed to proceed is already well described and tested. There are standardized tools readily available to practitioners to evaluate psychomotor development and help in conducting appropriate therapy, for instance, The General Movement Assessment (GMA), the Ages and Stages Questionnaire (ASQ-3), The Wechsler Preschool and Primary Scales of Intelligence (WPPSI-IV), and the Bayley Scales of Infant and Toddler Development (BSID-4). Follow-up visits should be widespread and mandatory in many countries to make opportunities more equal for all children at risk of developmental delay.

## Figures and Tables

**Table 1:** Typical developmental milestones at 4, 6, 9, and 12 months of age related to motor development, social-emotional skills, and infant communication abilities [14,15,16,17,18].

Age (Months)	Motor Development Milestones	Social and Emotional Milestones	Communication Milestones
4	Brings their hands to their mouth	Smiles spontaneously	Crying sounds different based on needs
	Rolls over onto their back	Tries copying movements and facial expressions	Begins to babble and copy sounds
	Holds their head up without support	Likes to play with others and may become upset when playtime stops	
	Shakes a toy while holding it		
6	Sits without support	Usually happy and responds to the emotions of others	Recognizes and responds to their name
	Rolls over in both directions	Starts to differentiate between familiar faces and strangers	Puts vowel sounds together and starts to emit some consonant sounds
	Pushes down on legs when feet are on a hard surface	Enjoys playing with you, and others	Responds to noises by making sounds and shows positive and negative emotions
	Rocks back and forth	Has fun looking at themself in the mirror	
9	Can move into a sitting position and sit without support	Starts to cling to familiar adults, and may be afraid of strangers	Points at objects with fingers
	Pulls themselves up to stand using furniture for support	Has favorite toys and reaches for them often	Understands the word “no,” and makes different sounds
	Starts to crawl		Starts to copy movements and sounds heard
12	Can take a few steps without support	May hand others a book when wanting to hear a story	Uses basic gestures such as waving and saying basic words like “mama” and “dada”
	Moves into a sitting position without support and pulls to stand	Cries when parents leave	Their babbles sound more like speech
	Walks while holding onto furniture	Is shy around strangers	Demonstrates responsiveness to straightforward commands
		Holds out his hand or leg to help dress himself	Attempts to repeat words spoken by others
		Has favorite toys	
		Repeats sounds to obtain attention	

**Table 2:** From the Lee SW, Koo MJ. PRISMA 2020 statement and guidelines for systematic review and meta-analysis articles, and their underlying mathematic Life Cycle Committee Recommendations. *Life Cycle* **2022**, *2*, e9. https://doi.org/10.54724/lc.2022.e9 [42].

Section and Topic	Item #	Checklist Item	Location Where Item Is Reported
**TITLE**			
Title	1	Identify the report as a systematic review.	N/A
**ABSTRACT**			
Abstract	2	See the PRISMA 2020 for Abstracts checklist.	N/A
**INTRODUCTION**			
Rationale	3	Describe the rationale for the review in the context of existing knowledge.	
Objectives	4	Provide an explicit statement of the objective(s) or question(s) the review addresses.	111–113 lines
**METHODS**			
Eligibility criteria	5	Specify the inclusion and exclusion criteria for the review and how studies were grouped for the syntheses.	126–147 lines
Information sources	6	Specify all databases, registers, websites, organisations, reference lists and other sources searched or consulted to identify studies. Specify the date when each source was last searched or consulted.	116–125 lines
Search strategy	7	Present the full search strategies for all databases, registers and websites, including any filters and limits used.	N/A
Selection process	8	Specify the methods used to decide whether a study met the inclusion criteria of the review, including how many reviewers screened each record and each report retrieved, whether they worked independently, and if applicable, details of automation tools used in the process.	126–147 lines
Data collection process	9	Specify the methods used to collect data from reports, including how many reviewers collected data from each report, whether they worked independently, any processes for obtaining or confirming data from study investigators, and if applicable, details of automation tools used in the process.	N/A
Data items	10a	List and define all outcomes for which data were sought. Specify whether all results that were compatible with each outcome domain in each study were sought (e.g., for all measures, time points, analyses), and if not, the methods used to decide which results to collect.	N/A
	10b	List and define all other variables for which data were sought (e.g., participant and intervention characteristics, funding sources). Describe any assumptions made about any missing or unclear information.	N/A
Study risk of bias assessment	11	Specify the methods used to assess risk of bias in the included studies, including details of the tool(s) used, how many reviewers assessed each study and whether they worked independently, and if applicable, details of automation tools used in the process.	N/A
Effect measures	12	Specify for each outcome the effect measure(s) (e.g., risk ratio, mean difference) used in the synthesis or presentation of results.	N/A
Synthesis methods	13a	Describe the processes used to decide which studies were eligible for each synthesis (e.g., tabulating the study intervention characteristics and comparing against the planned groups for each synthesis (item #5)).	126–147 lines
	13b	Describe any methods required to prepare the data for presentation or synthesis, such as handling of missing summary statistics, or data conversions.	N/A
	13c	Describe any methods used to tabulate or visually display results of individual studies and syntheses.	N/A
	13d	Describe any methods used to synthesize results and provide a rationale for the choice(s). If meta-analysis was performed, describe the model(s), method(s) to identify the presence and extent of statistical heterogeneity, and software package(s) used.	126–147 lines
	13e	Describe any methods used to explore possible causes of heterogeneity among study results (e.g., subgroup analysis, meta-regression).	N/A
	13f	Describe any sensitivity analyses conducted to assess robustness of the synthesized results.	N/A
Reporting bias assessment	14	Describe any methods used to assess risk of bias due to missing results in a synthesis (arising from reporting biases).	N/A
Certainty assessment	15	Describe any methods used to assess certainty (or confidence) in the body of evidence for an outcome.	126–147 lines
**RESULTS**			
Study selection	16a	Describe the results of the search and selection process, from the number of records identified in the search to the number of studies included in the review, ideally using a flow diagram.	Results section
	16b	Cite studies that might appear to meet the inclusion criteria, but which were excluded, and explain why they were excluded.	N/A
Study characteristics	17	Cite each included study and present its characteristics.	Results section
Risk of bias in studies	18	Present assessments of risk of bias for each included study.	N/A
Results of individual studies	19	For all outcomes, present, for each study: (a) summary statistics for each group (where appropriate) and (b) an effect estimate and its precision (e.g., confidence/credible interval), ideally using structured tables or plots.	N/A
Results of syntheses	20a	For each synthesis, briefly summarise the characteristics and risk of bias among contributing studies.	N/A
	20b	Present results of all statistical syntheses conducted. If meta-analysis was done, present for each the summary estimate and its precision (e.g., confidence/credible interval) and measures of statistical heterogeneity. If comparing groups, describe the direction of the effect.	N/A
	20c	Present results of all investigations of possible causes of heterogeneity among study results.	N/A
	20d	Present results of all sensitivity analyses conducted to assess the robustness of the synthesized results.	N/A
Reporting biases	21	Present assessments of risk of bias due to missing results (arising from reporting biases) for each synthesis assessed.	N/A
Certainty of evidence	22	Present assessments of certainty (or confidence) in the body of evidence for each outcome assessed.	126–147 lines
**DISCUSSION**			
Discussion	23a	Provide a general interpretation of the results in the context of other evidence.	180 line, 208–213 lines,
	23b	Discuss any limitations of the evidence included in the review.	N/A
	23c	Discuss any limitations of the review processes used.	N/A
	23d	Discuss implications of the results for practice, policy, and future research.	194–200, 220–221, 233–238 lines
**OTHER INFORMATION**			
Registration and protocol	24a	Provide registration information for the review, including register name and registration number, or state that the review was not registered.	N/A
	24b	Indicate where the review protocol can be accessed, or state that a protocol was not prepared.	N/A
	24c	Describe and explain any amendments to information provided at registration or in the protocol.	N/A
Support	25	Describe sources of financial or non-financial support for the review, and the role of the funders or sponsors in the review.	In Editorial System
Competing interests	26	Declare any competing interests of review authors.	In Editorial System
Availability of data, code and other materials	27	Report which of the following are publicly available and where they can be found: template data collection forms; data extracted from included studies; data used for all analyses; analytic code; any other materials used in the review.	N/A

**Table 3:** The contents of the results.

Studies Name	Year	The Quality of Evidence	Characteristic
National Guideline Alliance European Foundation for the Care of Newborn Infants American Academy of Pediatrics	2017 2018 2023	high	The National Guideline Alliance, the National Institute for Health and Care Excellence (NICE), the EFCNIs, and the AAP stated that children born before 32 weeks of gestation (GA) and term-born infants at risk for developing adverse neuromotor outcomes should have follow-up visits in the clinic and/or home to reassess any developmental concerns [13,42].
**Hadders-Algra M.** **Hielkema T et al.** **Janssen AJWM et al.** **Romeo DM et al.** **Romeo DM et al.** **Heineman KR et al.** **Barnett AL et al.** Hadders-Algra M.	2018 2020 2011 2022 2016 2008 2004 2005	high	All healthcare professionals should adhere to the periodic neuromotor assessment guidelines in the first two years of life (e.g., 3–6, 12, 24 months corrected age) and subsequently repeat this periodic assessment during the transition to school [24,29,31,43,44,45,46,47,48].
Surveillance of Cerebral Palsy in Europe **Atkins D et al.** **Gorter JW et al.** **Virella D et al.** **Smithers-Sheedy H et al.**	2001, 2023 2004 2009 2016 2014	high	For children with a diagnosis of CP, from 12 months of corrected age onwards, at each follow-up appointment, a standardized assessment of CP (according to the Surveillance of Cerebral Palsy in Europe (SCPE) criteria [49]) should be performed, and from 24 months of corrected age onwards, an assessment of the functional level of gross motor function, manual ability, and communication should be performed [50,51,52]. An early diagnosis of CP is the standard of care in many countries, and early intervention is possible to harness neuroplasticity [40]. More researchers are testing interventions with infants with CP due to the early and accurate identification of infants with CP. This is because current handling techniques for skill development during infancy are ineffective, and children with CP should receive proper intervention during this very critical time of brain development [53].
WHO Guidelines on Parenting Interventions to Prevent Maltreatment and Enhance Parent–child Relationships with Children Aged 0–17 Years **Fauls JR et al.** **Zubler J et al.**	2022 2020 2022	high	Standardized neuromotor assessments should be used from infancy until the time at which children start school (e.g., The General Movement Assessment (GMA) at 3–4 months of corrected age; the Parent Report of Children’s Abilities-Revised (PARCA-R) should be performed from two years of life with corrected age; and the Ages and Stages Questionnaire (ASQ) 48-month questionnaire, etc.) should be administered by qualified healthcare professionals [16,17,18]. The Wechsler Preschool and Primary Scales of Intelligence—4th Edition (WPPSI)—is recommended as a standardized test to assess IQ. Therefore, healthcare professionals should be properly trained to administer these assessments [13,54].

## Data Availability

The datasets used and analyzed during the current study are available from the corresponding author upon reasonable request.
